# Multi-omic profiles of human non-alcoholic fatty liver disease tissue highlight heterogenic phenotypes

**DOI:** 10.1038/sdata.2015.68

**Published:** 2015-12-08

**Authors:** Wasco Wruck, Karl Kashofer, Samrina Rehman, Andriani Daskalaki, Daniela Berg, Ewa Gralka, Justyna Jozefczuk, Katharina Drews, Vikash Pandey, Christian Regenbrecht, Christoph Wierling, Paola Turano, Ulrike Korf, Kurt Zatloukal, Hans Lehrach, Hans V. Westerhoff, James Adjaye

**Affiliations:** 1 Medical Faculty, Institute for Stem Cell Research and Regenerative Medicine, Heinrich Heine University, 40225 Düsseldorf, Germany; 2 Institute of Pathology, Medical University of Graz, Graz 8036, Austria; 3 The Manchester Centre for Integrative Systems Biology, Manchester Institute of Biotechnology, University of Manchester, Manchester M1 7DN, UK; 4 Department of Vertebrate Genomics, Max Planck Institute for Molecular Genetics, Ihnestrasse 63, Berlin 14195, Germany; 5 Division of Molecular Genome Analysis, German Cancer Research Center (DKFZ), 69120 Heidelberg, Germany; 6 Magnetic Resonance Center (CERM), University of Florence, 50019 Florence, Italy; 7 Institute for Pathology & Comprehensive Cancer Center, Cancer Stem Cell Group, Charité—Universitätsmedizin, 10117 Berlin, Germany; 8 Netherlands Institute for Systems Biology, VU University Amsterdam, HV NL-1081 Amsterdam, The Netherlands; 9 Synthetic Systems Biology, Swammerdam Institute for Life Sciences, University of Amsterdam, 1018 WS Amsterdam, The Netherlands

**Keywords:** Non-alcoholic fatty liver disease, Metabolomics, Gene expression analysis, Systems biology

## Abstract

Non-alcoholic fatty liver disease (NAFLD) is a consequence of sedentary life style and high fat diets with an estimated prevalence of about 30% in western countries. It is associated with insulin resistance, obesity, glucose intolerance and drug toxicity. Additionally, polymorphisms within, e.g., *APOC3, PNPLA3, NCAN, TM6SF2* and *PPP1R3B*, correlate with NAFLD. Several studies have already investigated later stages of the disease. This study explores the early steatosis stage of NAFLD with the aim of identifying molecular mechanisms underlying the etiology of NAFLD. We analyzed liver biopsies and serum samples from patients with high- and low-grade steatosis (also pre-disease states) employing transcriptomics, ELISA-based serum protein analyses and metabolomics. Here, we provide a detailed description of the various related datasets produced in the course of this study. These datasets may help other researchers find new clues for the etiology of NAFLD and the mechanisms underlying its progression to more severe disease states.

## Background & Summary

With an estimated prevalence of about 30% in western countries, NAFLD is a major public health issue^1^. Sedentary life-style and excessive food consumption correlate with rate at which NAFLD cases appear. Epidemiologic studies showing a prevalence of the disease that differs between countries as well as between groups in the same country, appear to reflect an interplay of environmental and genetic factors in its etiology^1^. Additionally, polymorphisms in, e.g., *APOC3, PNPLA3, NCAN, TM6SF2 and PPP1R3B*, correlate with NAFLD^2,3^. Over-feeding directly induces insulin resistance^4^. Causality between steatosis and the metabolic syndrome of insulin resistance, obesity, and glucose intolerance, is still unresolved^5^. While the correlation between steatosis and insulin resistance is established there is debate about the relationship between steatosis and hepatic insulin resistance^6^. Samuel *et al.* showed that activated *PKC-ϵ* and *JNK* can induce insulin resistance via impaired *IRS1* and *IRS2* tyrosine phosphorylation in rats fed with high fat diet^7^. An investigation on the insulin-like growth factor (IGF) axis in the Nurses’ Health Study^8^ and another population study of 3863 people^9^ addressed connections between the IGF axis, insulin resistance, diabetes risk and NAFLD. *IGFBP3* is associated with various cancers and up-regulation of *IGF1* receptor (*IGF1R*) is considered an early event in hepatocarcinogenesis^10^. Thus, the IGF axis might play an important role in a direct development of carcinoma from steatosis without the formerly assumed intermediary phase of cirrhosis^11^.

The progression of NAFLD from mild steatosis up to severe steatohepatitis and even liver cirrhosis and hepatocellular carcinoma, varies widely between individual patients. Insulin resistance, dysregulation of cytokines as a basis for inflammation, and oxidative stress appear to foster progression to steatohepatitis^12^. A two-step progression from simple steatosis to steatohepatitis and fibrosis has been proposed^13^, and suggests that after fat accumulation in the liver due to insulin resistance, lipids are peroxidized with cytokines and Fas ligand induced by excessive ROS. However, this two-step progression has been questioned^5^. We found that in fibroblasts derived from steatosis patients *AKT/mTOR* signaling was reduced and that the insulin-resistant phenotype is exhibited not only by insulin-metabolizing central organs, e.g., the liver, but also by skin fibroblasts^14^. Transcriptome data identified a regulatory network orchestrated by the transcription factor *SREBF1* and linked to a metabolic network of glycerolipid and fatty acid biosynthesis. The downstream transcriptional targets of *SREBF1* which include the phosphatidic acid phosphatase *LPIN1* and *LDLR*, were also involved.

Moreover, there is the possible involvement of ROS in disease progression. Houstis *et al.* ^15^ demonstrated that oxidative stress can induce insulin resistance and that anti-oxidants may ameliorate insulin resistance. Depletion of glutathione can improve insulin sensitivity in mice^16^. Glutathione is known as the body’s master antioxidant, protecting cells against damage caused by numerous reactive intermediates^17^. Detoxification of these reactive metabolites results in the consumption of glutathione either via oxidation or conjugation. Maintenance of the intracellular glutathione level is thereby a critical liver function, which could be impaired following insult/injury or in steatosis and steatohepatitis.

Several other studies exploring various aspects of NAFLD have been published. A recent publication by Moylan *et al.* showed that it is possible to discriminate mild versus severe fibrosis stages of NAFLD patients via their gene expression profiles^18^. Another study from Speliotes *et al.* investigated NAFLD via a genome-wide association study (GWAS) approach^3^. Besides the most prominent association of *PNPLA3* this study reported several other associations including one at locus 19p13.11 which is in strong linkage disequilibrium with a recently found steatosis-linked polymorphism in *TM6SF2*, transmembrane6 superfamily member 2 (refs 19,^20^) . A knockdown of TM6SF2 in human hepatoma cell lines and in mice led to an increase in lipid droplet area while overexpression led to a decrease^19^.

Interestingly, the above mentioned genes associated with NAFLD in GWAS were not detected in a large-scale GWAS about obesity and insulin biology although the metabolic syndrome connects NAFLD and obesity^21^. Feldstein *et al.* found CK-18 as a non-invasive biomarker for NASH by comparison of plasma samples from patients with biopsy proven NAFLD^22^. Du Plessis *et al.* used analysis results from subcutaneous and visceral fat and liver biopsies to construct a model which predicts NAFLD liver histology^23^. This model involves the genes *CCL2, DMRT2, GADD45B, IL1RN*, and *IL8.* In contrast to the studies of Moylan *et al.* and Feldstein *et al.* our study highlights potential means of classifying distinct grades of Steatosis in NAFLD—the very early stage of the disease. Although it is evident that a complex interplay of genetic and environmental factors contribute to the development of steatosis, to date there has not been a systemic study of the disease employing a multi-omic approach- transcriptome, ELISA-based proteome and metabolome. Therefore, the intention of this study is to provide a more comprehensive view of steatosis based on transcriptomic, metabolomic and protein biomarker profiles. Additionally, this should lay down the foundation for follow-up systems biology-based studies.

In the current study we analyzed patient liver biopsies and associated serum samples, from patients with the insulin resistance phenotype confirmed by the HOMA-IR model^24^. Here, we describe these valuable data sets deposited in public repositories, which might support other researchers in identifying new clues for the etiology of NAFLD and the mechanisms underlying its progression to more severe disease states.

## Methods

### Patient recruitment, sample collection and clinical measurements

**Figure 1:**
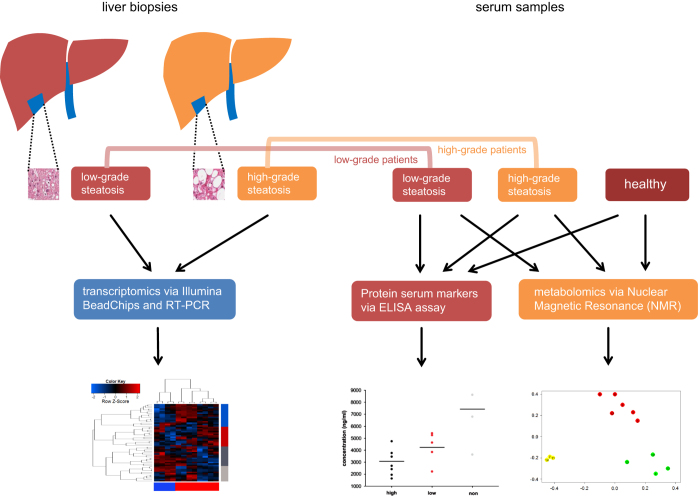
Scheme of experiments for multi-omics comparison of steatosis grades. The scheme shows how the distinct severities of non-alcoholic fatty liver disease (NAFLD) are compared in terms of transcriptomics, metabolomics and potentially relevant parts of the proteome. Liver biopsies were taken from NAFLD patients and classified by pathologists as low-grade (5–33% steatosis area) and high-grade (>33% steatosis area). The transcriptome of liver biopsies were assessed on Illumina HumanHT-12 v4 BeadChips and on RT-PCR. Serum samples of these NAFLD patients and from healthy persons were taken and investigated at the protein level employing ELISA assays and at the metabolome level via Nuclear Magnetic Resonance (NMR).

**Figure 2:**
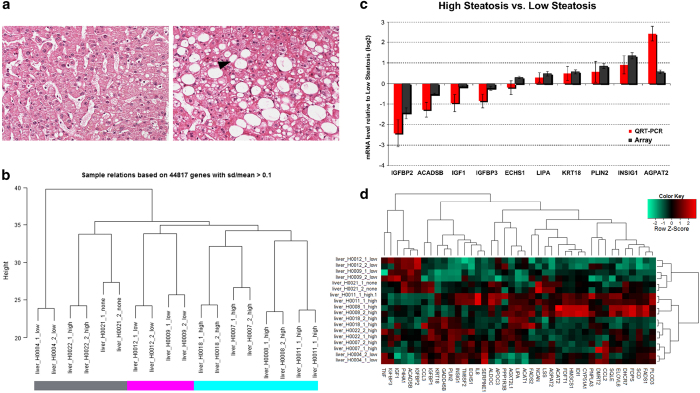
Histopathological and transcriptome characterization of liver tissue. (**a**) Liver tissue with only marginal pathological changes (H9, low-grade steatosis group). The hepatocytes are arranged in one cell thick plates, separated by sinusoids. They contain only few small isolated fat valuoles (H&E stained section). Hepatocytes of the intermediate and central lobular areas contain macrovesicular fat (image to the right, H8, steatosis group, hepatocytes with fatty change are indicated by arrow heads; H&E stained section). (**b**) Hierachical clustering of the transcriptomes of patient liver samples. We identified three clusters: high (>33%) steatosis (cyan), low (5–33%) steatosis (magenta) and heterogeneous clusters of high, low and no steatosis (grey). (**c**) Quantitative QRT-PCR confirmation of genes differentially expressed in high versus low steatotic livers. The columns represent the mean of four biological replicates (high steatosis) versus two biological replicates (low steatosis). Error bars indicate standard errors of the mean. Array-derived and RT-PCR-derived columns are depicted in dark grey and red respectively. (**d**) Heatmap of genes differentially expressed in high versus low steatotic livers and genes found in literature and in genome-wide association studies.

All patients participating in this study were recruited in the Multidisciplinary Obesity Research (MORE) project at the Medical University of Graz, Austria or at the Interdisciplinary Adipositas Center at the Kantonsspital St Gallen, Switzerland. Patients with morbid obesity who admitted into hospital for treatment by bariatric surgery (gastric banding, gastric bypass, sleeve gastrectomy) were invited to participate in the study and to sign the informed consent. The study was approved by the institutional review board of the Medical University of Graz (reg. IRB00002556 at the Office for Human Research Protections of the US Departments of Health and Human Services) under license 20–143 ex 08/09. All experiments were performed in accordance with approved guidelines. Written informed consent was obtained from all participants. In the course of the bariatric surgery, samples of blood, skin and a liver biopsy were taken. Out of 18 patients (Table 1), 9 liver biopsies were of high quality enabling their use in the transcriptome analyses. Serum plasma was available from all the patients. The overall experimental design of this study is illustrated in Fig. 1. A pathological diagnosis of the liver phenotype, including liver steatosis grading based on H&E morphology, was performed by an experienced, board certified pathologist (CL). We simplified Kleiner’s scoring scheme by condensing Steatosis grades 2 (34–66%) and 3 (> 66%) to our ‘high-grade’ while adopting grades 0 (‘none’) and 1 (‘low’)^25^. This simplification was made because the inter-patient-variability in this complex heterogeneous disease did not allow a more detailed grading on the omics levels. Two examples of liver biopsies are shown in Fig. 2a.

### Illumina bead chip hybridization and data analysis

Microarray experiments were carried out on the Illumina BeadStation 500 platform (Illumina, San Diego, CA, USA). Briefly, 500 ng DNase-treated total RNA were used as input for amplification and biotin labeling reactions (Illumina TotalPrep RNA Amplification Kit, Ambion) prior to hybridization of the resulting cRNAs onto Illumina HumanHT-12_v4_BeadChips, washing, Cy3-streptavidin staining and scanning according to the manufacturer’s instructions.

### Transciptomics data analysis

Illumina data was processed via R/Bioconductor^26^ and packages lumi^27^, limma^28^ and biomaRt. Background-corrected log2-transformed data was normalized via quantile normalization from the lumi package.

### qRT-PCR

Quantitative real-time polymerase chain reaction (qRT-PCR) was carried out to confirm the microarray-derived data. Reactions were carried out on the ABI PRISM 7900HT Sequence Detection System (Applied Biosystems). Data analysis was carried out using the ABI PRISM SDS 2.2.1 software (Applied Biosystems) and Microsoft Excel (Microsoft Corporation, Redmond, WA, USA). GAPDH-normalized, relative mRNA levels of each gene (high steatosis versus low steatosis) were calculated based on the 2-ΔΔCT Method. Primer sequences for QRT-PCR validation are described in Table 2.

### ELISA-based assay for biomarkers

ELISA measurements from plasma samples were carried out using the Ciraplex platform (Aushon Biosystems, Billerica, MA, US). Commercial assays were purchased and measurements were carried out according to instructions provided by the manufacturer. The following 29 targets were analyzed either as single-plex assays or as multiplex assay: hFGFb; hGROa; hLIF;hIFNg; hIL1b; IL4; IL5; hIL6; hIL10; hIL12p70; hIL13; hTNFa; hI309; hIL8; hIP10; hMCP4; hMIP1a; hMIP1b; hCRP; hLeptin; hPAIactive; hResistin; hIGFBP1; hIGFBP3; hIGFBP2; hMIF; hApoA1; hCRP; hAcrp30.

### NMR sample preparation

Frozen plasma samples were thawed at room temperature and shaken before use. According to standard methodologies a total of 300 μl of buffer (70 mM Na2HPO4; 20% (v/v) D2O; 6.15 mM NaN3; 6.64 mM TMSP; pH 7.4) was added to 300 μl of each serum sample. A total of 450 μl of this mixture was transferred into a 4.25 mm NMR tubes (Bruker BioSpin) for analysis.

### NMR spectra acquisition and processing

NMR spectra from 18 plasma samples from morbidly obese patients that underwent different type of bariatric surgery and additionally have developed steatosis were collected (Table 1). 1H-NMR spectra were acquired using a Bruker spectrometer (Bruker Biospin). Unsupervised and supervised methods were used in order to identify a disease-related metabolomic profile that might contain a signature of steatosis.

## Data Records

### Data record 1

The microarray experiments discussed in this publication were carried out on the Illumina BeadStation 500 platform (Illumina, San Diego, CA, USA). The data have been deposited in NCBI's GEO and are accessible through GEO Series accession number GSE46300 (Data Citation 1).

### Data record 2

Metabolomic raw data from Nuclear magnetic resonance (NMR) measurements have been deposited at the MetaboLights database (http://www.ebi.ac.uk/metabolights) of the European Bioinformatics Institute (EBI) under MTBLS174 (Data Citation 2).

### Data record 3

ELISA measurements have been deposited at figshare (http://www.figshare.com) (Data Citation 3).

## Technical Validation

### Transcriptomic data

**Figure 3:**
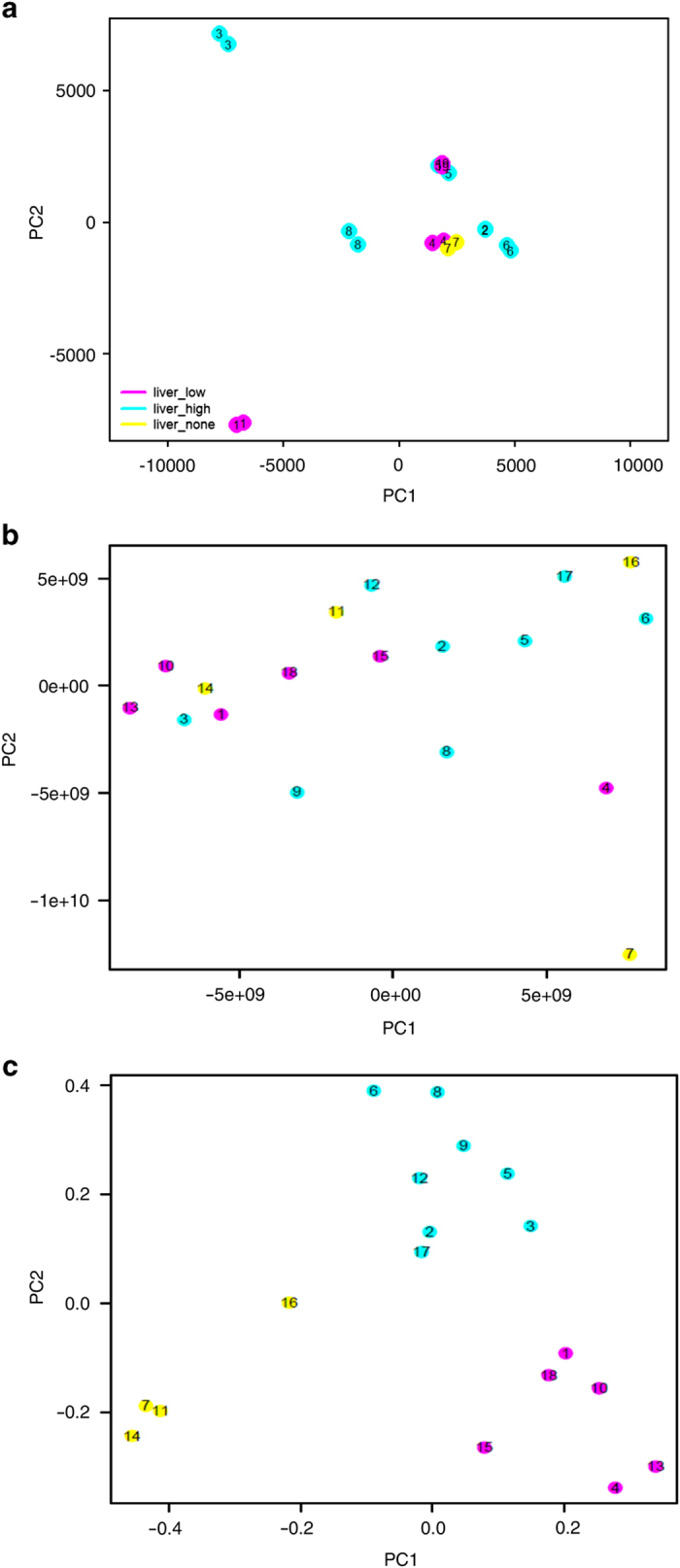
Transcriptomic and metabolomic profiles. (**a**–**c**) Transcriptomics and metabolomics PCA plots. Distinct colours are used to aid visualizing patients with distinct levels of steatosis: yellow, patients with <5% no steatosis; magenta, patients with 5–33%, low level steatosis; cyan, patients with high steatosis >33%, high steatosis. (**a**) Unsupervised PCA plot for 18 liver biopsies, Illumina microarray data. (**b**) Unsupervised PCA plot for 18 plasma samples, metabolomics data. (**c**) Supervised discrimination analysis (pls/ca: partial least squares/canonical analysis) of metabolites in patient plasma samples. The correspondence between numbers in the plot and sample names in Table 1 is: 1=H0004, 2=H0007, 3=H0008, 4=H0009, 5=H0011, 6=H0018, 7=H0021, 8=H0022, 9=H0024, 10=H0025, 11=H0026, 12=H0027, 13=H0028, 14=H0029, 15=H0030, 16=H0031, 17=H0033, 18=H0034, 19=H0012.

Microarray data passed the proprietary Illumina quality controls. All samples were investigated in duplicates. Fig. 2b shows that—as would be expected—the duplicates cluster together demonstrating the validity of experiments in terms of whole-genome gene expression. The Pearson correlation coefficients of all samples versus each other were calculated with the intention to detect outliers. However, all correlation coefficients were greater than 0.98 and all correlation coefficients of duplicates even greater than 0.99 so that all samples passed this quality check (Table 3). Genes with significant differential gene expression were selected for validation via RT-PCR experiments (Fig. 2c). Genes were termed differentially expressed if the multiple-testing-corrected limma^28^ *P*-value was less than 0.05, the ratio was less than 0.75 or greater than 1.33 and the gene was expressed (detection *P*-value less than 0.05) in at least one of both cases. Furthermore, we analysed clusters of genes differentially expressed in high versus low steatotic livers together with genes found in literature^19,23^ and genome-wide association studies^3^ (Fig. 2d). This analysis confirms high similarity between duplicates and clustering—to some extent but not fully—according to steatosis grade. Fig. 3a shows a plot of the first two components of the Principal Component Analysis (PCA) of the microarray data.

### ELISA-based assay for biomarkers

Samples below and above quantification limit as well as samples with coefficient of variation (cv) greater than 20% were marked in the measurements table (Data Citation 3). Independent validation of the ELISA-based measurements was checked by visual inspection of plots comparing disease states.

### Metabolomics

Assignment of all metabolites were done manually, signals were assigned on template one-dimensional NMR profiles by using matching routines of AMIX 7.3.2 (Bruker BioSpin) in combination with the BBIOREFCODE Version 2-0-0 reference database and published literature when available. Additional confirmation was done using data provided in our lab -database containing spectra of standard pure compounds. To assess which metabolites (i.e., NMR peaks) were significantly different between different sets a univariate paired Wilcoxon test was used. A *P*-value≤0.05 was considered statistically significant (*P*-value not corrected for multiple testing).

Robust validation of statistical analysis results was done using a cross-validation technique.

The accuracy of the classification was assessed by means of a single cross-validation scheme. The original data set was split into a training set (80% of the samples) and a test set (20% of the samples) prior to any step of statistical analysis. The number of PLS components was chosen on the basis of a 5-fold cross validation performed on the training set only, and the best model was used to predict the samples in the test set. The whole procedure was repeated 200 times with a Monte Carlo cross validation scheme, and the results averaged.

Figure 3b shows a plot of unsupervised discrimination analysis and Fig. 3c shows separation of steatosis grades in a plot of supervised discrimination analysis (pls/ca: partial least squares/canonical analysis) of metabolites in patient plasma samples. The clustering of Fig. 3c results from a supervised PLS/CA based only on the metabolomic NMR profiles. The algorithm takes into account the supervised information relative to the 3 steatosis groups.

### Distribution plots

**Figure 4:**
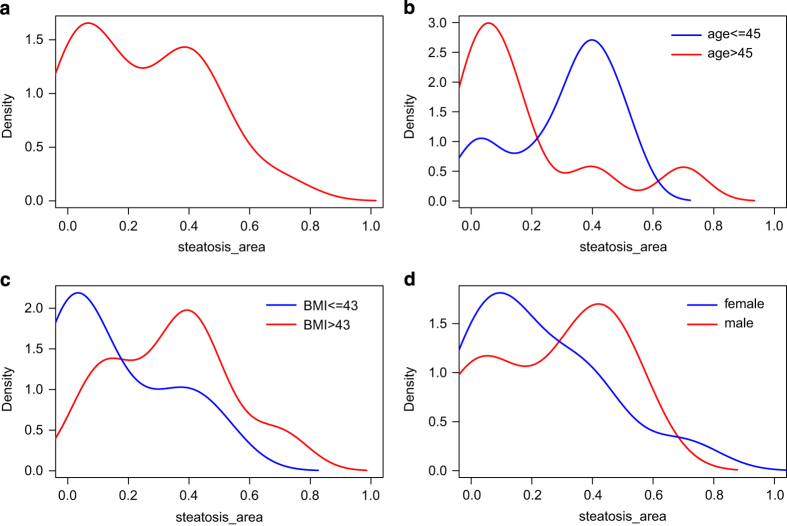
Distribution plots of percentage parenchymal involvement in steatosis. (**a**) all patients. (**b**) Kernel density plot of patients above/below median age (median=45). (**c**) Kernel density plot of patients above/below median BMI (median=43). (**d**) Kernel density plot of male/female patients.

Figure 4a shows the distribution of percentage parenchymal involvement in steatotic patients derived from Table 1. The percentage is converted to a scale from zero to one and plotted with the kernel density function from the R statistical package. Fig. 4b–d display distributions separated into groups of age above/below median (median=45), body mass index (BMI) above/below median (median=43) and gender. Fig. 4d would suggest a slight tendency for more severe steatosis in males. A similar trend has been reported in a NAFLD study on Australian adolescents where 3.1% of males and only 2.2% of females had moderate to severe steatosis while 7.0% of males and 14.1% of females had mild steatosis^29^.

## Usage Notes

All patients in this study underwent bariatric surgery. This should be taken into account when generalizing results although these are typical cases of morbid obesity which is connected to the metabolic syndrome including NAFLD. The sample size of these datasets—in particular the transcriptomics dataset—poses certain limits onto its usage. Due to its small size it will not enable rigorous analysis of gender effects. Therefore it would likely need to be combined with other data sources, such as data from Moylan *et al.*^18^ and Du Plessis *et al.*^23^.

## Additional Information

**How to cite this article:** Wruck, W. *et al.* Multi-omic profiles of human non-alcoholic fatty liver disease tissue highlight heterogenic phenotypes. *Sci. Data* 2:150068 doi: 10.1038/sdata.2015.68 (2015).

## Supplementary Material



## Figures and Tables

**Table 1:** Samples related to data sets in repositories (Data Citations 1–Data Citation 3).

**ID**	**gender**	**Age**	**BMI**	**% steatosis**	**grouping by pathologist**	**steatosis grouping**	**medical centre**	**liver illumina array rep.1 (GSE46300)**	**liver illumina array rep.2 (GSE46300)**	**serum NMR data**	**serum ELISA data**
H0004	f	54	47	10%		obese, low steatosis	Graz (Austria)	GSM1128362	GSM1128363	MTBLS174	10.6084/m9.figshare.1333564
H0007	f	33	51	40%		obese, high steatosis	Graz (Austria)	GSM1128364	GSM1128365	MTBLS174	10.6084/m9.figshare.1333564
H0008	m	61	46	40%	obese, high steatosis	obese, high steatosis	Graz (Austria)	GSM1128366	GSM1128367	MTBLS174	10.6084/m9.figshare.1333564
H0009	f	48	49	5–10%	obese, low steatosis	obese, low steatosis	Graz (Austria)	GSM1128368	GSM1128369	MTBLS174	10.6084/m9.figshare.1333564
H0011	f	58	45	70%	obese, high steatosis	obese, high steatosis	Graz (Austria)	GSM1128370	GSM1128371	MTBLS174	10.6084/m9.figshare.1333564
H0012	f	50	35	0	obese, low steatosis	obese, low steatosis	Graz (Austria)	GSM1128372	GSM1128373	no	no
H0018	f	35	41	30–40%	obese, high steatosis	obese, high steatosis	Graz (Austria)	GSM1128374	GSM1128375	MTBLS174	10.6084/m9.figshare.1333564
H0021	m	49	41	0%		no steatosis	Graz (Austria)	GSM1128376	GSM1128377	MTBLS174	10.6084/m9.figshare.1333564
H0022	m	45	49	40%		obese, high steatosis	Graz (Austria)	GSM1128378	GSM1128379	MTBLS174	10.6084/m9.figshare.1333564
H0024	m	29	44	50%		obese, high steatosis	Graz (Austria)	no	no	MTBLS174	10.6084/m9.figshare.1333564
H0025	f	53	46	15–20%		obese, low steatosis	St Gallen (Switzerland)	no	no	MTBLS174	10.6084/m9.figshare.1333564
H0026	f	46	39	0%		no steatosis	St Gallen (Switzerland)	no	no	MTBLS174	10.6084/m9.figshare.1333564
H0027	m	44	42	50%		obese, high steatosis	St Gallen (Switzerland)	no	no	MTBLS174	10.6084/m9.figshare.1333564
H0028	f	28	43	20%		obese, low steatosis	St Gallen (Switzerland)	no	no	MTBLS174	10.6084/m9.figshare.1333564
H0029	f	40	39	<5%		no steatosis	St Gallen (Switzerland)	no	no	MTBLS174	10.6084/m9.figshare.1333564
H0030	m	22	45	30%		obese, low steatosis	St Gallen (Switzerland)	no	no	MTBLS174	10.6084/m9.figshare.1333564
H0031	m	22	41	0%		no steatosis	St Gallen (Switzerland)	no	no	MTBLS174	10.6084/m9.figshare.1333564
H0033	f	44	43	40%		obese, high steatosis	St Gallen (Switzerland)	no	no	MTBLS174	10.6084/m9.figshare.1333564
H0034	m	50	42	10%		obese, low steatosis	St Gallen (Switzerland)	no	no	MTBLS174	10.6084/m9.figshare.1333564

**Table 2:** Primer sequences for QRT-PCR validation of genes differentially expressed between high-grade and low-grade steatosis.

**Gene**	**Fwd**	**Rev**	**Product size**
*ACADSB*	CACCATTGCAAAGCATATCG	GCAAGGCACTTACTCCCAAC	117
*AGPAT2*	GGGGCGTCTTCTTCATCA	TTGAGGTTCTCCCTGACCAT	91
*ECHS1*	AACCTTTGCCACTGATGACC	CAAGCAGAGGTGTGAAGCAG	112
*IGF1*	TGCAGGAGGGACTCTGAAAC	AGCTGCGTGATATTTGAAAGG	111
*IGFBP2*	CTCCCTGCCAACAGGAACTG	TCTTGCACTGTTTGAGGTTGTACAG	147
*IGFBP3*	CAACTGTGGCCATGACTGAG	CCTGACTTTGCCAGACCTTC	92
*INSIG1*	CAACACCTGGCATCATCG	CTCGGGGAAGAGAGTGACAT	118
*KRT18*	GAGGTTGGAGCTGCTGAGAC	CAAGCTGGCCTTCAGATTTC	99
*LIPA*	CATCTGTGTGAAGCCAAAGC	AATCCCTGAGCTGAGTTTGC	112
*PLIN2*	GCTGAGCACATTGAGTCACG	TGGTACACCTTGGATGTTGG	102

**Table 3:**
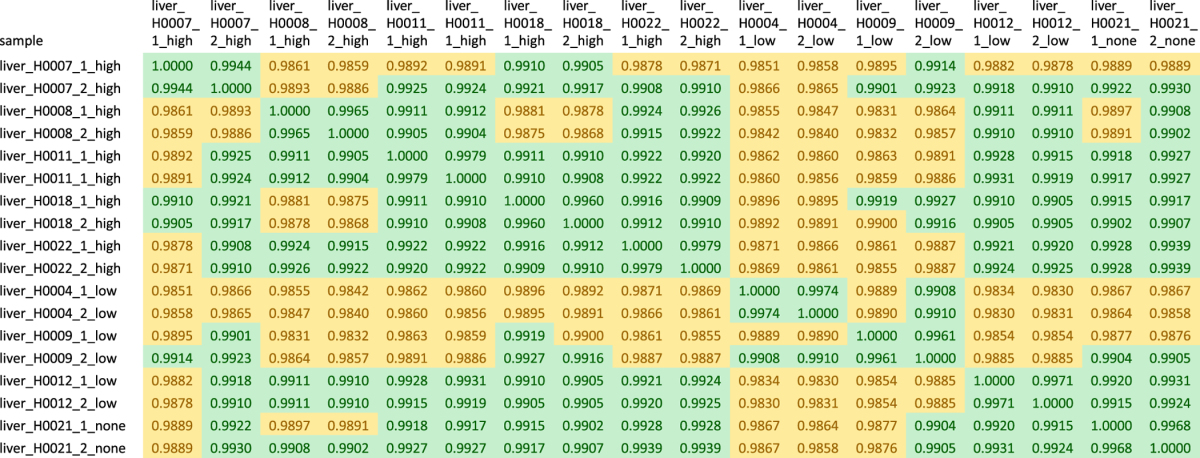
Pearson correlation coefficients of transcriptome data of all samples versus each other.
